# Macrophage polarization markers in subcutaneous, pericardial, and epicardial adipose tissue are altered in patients with coronary heart disease

**DOI:** 10.3389/fcvm.2023.1055069

**Published:** 2023-03-02

**Authors:** Bianca Papotti, Trine Baur Opstad, Sissel Åkra, Theis Tønnessen, Bjørn Braathen, Charlotte Holst Hansen, Harald Arnesen, Svein Solheim, Ingebjørg Seljeflot, Nicoletta Ronda

**Affiliations:** ^1^Department of Cardiology, Center for Clinical Heart Research, Oslo University Hospital Ullevål, Oslo, Norway; ^2^Department of Food and Drug, University of Parma, Parma, Italy; ^3^Faculty of Medicine, University of Oslo, Oslo, Norway; ^4^Department of Cardiothoracic Surgery, Oslo University Hospital, Oslo, Norway

**Keywords:** coronary heart disease, adipose tissue, macrophage polarization, inflammation, LDL-C, body mass index

## Abstract

**Background:**

Epicardial and pericardial adipose tissue (EAT and PAT) surround and protect the heart, with EAT directly sharing the microcirculation with the myocardium, possibly presenting a distinct macrophage phenotype that might affect the inflammatory environment in coronary heart disease (CHD). This study aims to investigate the expression of genes in different AT compartments driving the polarization of AT macrophages toward an anti-inflammatory (L-Galectin 9; CD206) or pro-inflammatory (NOS2) phenotype.

**Methods:**

EAT, PAT, and subcutaneous (SAT) biopsies were collected from 52 CHD patients undergoing coronary artery bypass grafting, and from 22 CTRLs undergoing aortic valve replacement. L-Galectin9 (L-Gal9), CD206, and NOS2 AT gene expression and circulating levels were analyzed through RT-PCR and ELISA, respectively.

**Results:**

L-Gal9, CD206, and NOS2 gene expression was similar in all AT compartments in CHD and CTRLs, as were also L-Gal9 and CD206 circulating levels, while NOS2 serum levels were higher in CHD (*p* = 0.012 vs. CTRLs). In CTRLs, NOS2 expression was lower in EAT vs. SAT (*p* = 0.007), while in CHD patients CD206 expression was lower in both SAT and EAT as compared to PAT (*p* = 0.003, *p* = 0.006, respectively), suggestive of a possible macrophage reprogramming toward a pro-inflammatory phenotype in EAT. In CHD patients, NOS2 expression in SAT correlated to that in PAT and EAT (*p* = 0.007, both), CD206 expression correlated positively to L-Gal9 (*p* < 0.001) only in EAT, and CD206 expression associated with that of macrophage identifying markers in all AT compartments (*p* < 0.001, all). In CHD patients, subjects with LDL-C above 1.8 mmol/L showed significantly higher NOS2 expression in PAT and EAT as compared to subjects with LDL-C levels below (*p* < 0.05), possibly reflecting increased cardiac AT pro-inflammatory activation. In SAT and PAT, CD206 expression associated with BMI in both CHD and CTRLs (*p* < 0.05, all), and with L-Gal9 in EAT, however only in CTRLs (*p* = 0.002).

**Conclusion:**

CHD seems to be accompanied by an altered cardiac, and especially epicardial AT macrophage polarization. This may represent an important pathophysiological mechanism and a promising field of therapy targeting the excessive AT inflammation, in need of further investigation.

**GRAPHICAL ABSTRACT:**
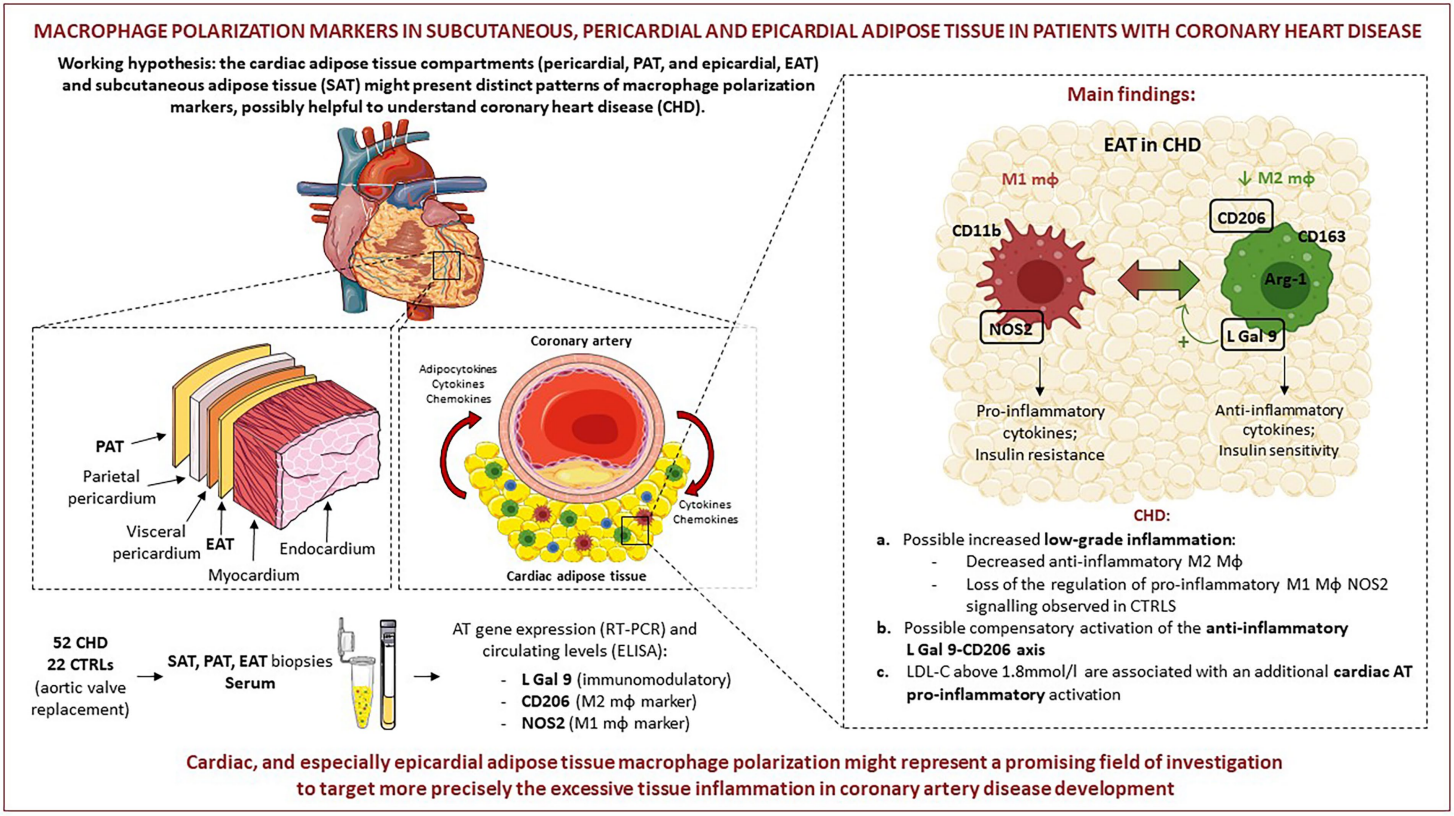
Pictures were created by combining images from Smart Servier Medical Art (https://smart-servier.com, accessed on 20.09.22). Servier Medical Art by Servier is licensed under a Creative Commons Attribution 3.0 Unported License (https://creativecommons.org/licenses/by/3.0/).

## 1. Introduction

The main cause of coronary heart disease (CHD) is atherosclerosis (1), where chronic inflammatory processes and obesity play pivotal roles. The heart is surrounded by adipose tissue (AT) that is both epicardial (EAT) and pericardial (PAT), with EAT embryonically originating from the splanchnopleuric mesoderm and PAT from thoracic mesenchyme (2). Anatomically, EAT is localized between the myocardium and the visceral pericardium and covers about 80% of the heart surface (3). It shares the microcirculation with the myocardial tissue and secretes various molecules through paracrine and “vasocrine” (4) mechanisms that may protect coronary arteries (3, 5, 6). PAT is separated from the heart by pericardium and, differently from EAT, receives blood supply from non-coronary arteries. Its role as a source of cardiac biochemical mediators is still a matter of debate (7). EAT exerts various anti-atherogenic and anti-inflammatory activities and regulates cardiac thermogenesis (8), while PAT apparently has less anti-inflammatory activity (9). Disturbances in AT composition and extension associate to adipocytes’ hypertrophy, insulin resistance and pro-inflammatory processes (10). According to some studies, the EAT volume is increased in coronary artery disease (CAD) patients (11, 12), although this was not confirmed by others (13), and may be a predictor of cardio-metabolic risk (14). Similarly, also increased PAT volume has been shown to associate with CV risk, lipid disorders, hypertension and obesity (7, 9). AT secretes a wide variety of adipokines, cytokines and chemokines (15, 16), thus being at the crossroad between metabolism and immunity. In addition to adipocytes, various resident immune cell populations can be found in AT, including adipose tissue macrophages (ATMs) (17), characterized by an high degree of phenotypic plasticity (17). Based on their inflammatory profile, ATMs may be classified, like other macrophages, as M1 pro-, and M2 anti-inflammatory elements (18). It is well accepted that ATMs in the tissues of lean subjects mainly exhibit the so-called alternatively activated M2 profile, releasing anti-inflammatory cytokines, known to impact positively on insulin sensitivity, angiogenesis and tissue repair (19). On the contrary, in obese subjects, ATMs are mainly polarized toward a classically activated M1 profile, known to produce pro-inflammatory cytokines, capable of inhibiting the normal insulin signaling in adipocytes (20). However, it is well accepted that macrophage polarization *in vivo* is a highly dynamic process that goes beyond the simplified M1/M2 classification (18). At this regard, Hirata and co-workers reported that in patients with CAD, EAT has an altered M1/M2 polarization (21), suggesting that the ATM phenotype may have an impact on cardiovascular disease (CVD), but to date the studies assessing potential differences between macrophage polarization in the different cardiac ATs are still limited.

The present study aims at investigating the polarization status of ATMs in EAT, PAT, and subcutaneous AT (SAT) from patients with CHD and from control patients with aortic valvular disease, to explore possible alterations specific to CHD and for each AT type. To this purpose, we measured gene expression of the following markers in the different cardiac AT compartments: (I) L-Galectin 9 (L-Gal 9), mainly produced in AT by macrophages and T cells (22), promoting anti-inflammatory T-regulatory cell activity and macrophage polarization toward an anti-inflammatory phenotype (23, 24); (II) CD206, a cell marker widely used to identify M2 macrophages (21, 25); and (III) nitric oxide synthase 2 (NOS2), a biomarker tightly connected with M1 macrophages, being transcribed following pro-inflammatory stimuli and involved in the effector molecule nitric oxide (NO) biosynthesis (26). Gene expression of the markers was related to their circulating protein levels and to patient clinical characteristics, including lipid profile and anthropometrics. In parallel, the expression of cell markers specific for macrophages, T lymphocytes and endothelial cells was also evaluated. Our hypothesis was that the different cardiac AT compartments might present a distinct pattern of macrophage polarization markers, with possible impact on CHD development.

## 2. Materials and methods

### 2.1. Patients

Fifty-two patients with CHD undergoing coronary artery bypass grafting and 22 control subjects (CTRLs) undergoing aortic valve replacement without evidence of CHD were recruited from December 2016 to May 2018 at Oslo University Hospital, Ullevål, Oslo (Norway). Before surgery, all patients gave written informed consent and the experimental protocol was approved by the Regional Ethics Committee of North Norway (#2016/411), following the Declaration of Helsinki. The inclusion criterium for the pathological condition under study was the indication for coronary artery bypass surgery due to CHD; with no specific restrictions to inclusion, except for lack of consent to participate to the study and the use of medications known to overtly interfere with inflammation (e.g., steroids). The study is registered at clinicaltrials.gov with the code NCT02760914. Briefly, during the open-chest surgery and before starting the extracorporeal circulation, representative biopsies (approximately 0.5–1.5 cm) from SAT (pre-sternally at the middle of the sternum), PAT (ventrally to the pericardium next to the aorta) and EAT (area between the right coronary artery and the pulmonary artery) were isolated and immediately deep-frozen at −80°C until RNA extraction. Before anesthesia, arterial blood samples were collected. More details have previously been published (27).

### 2.2. Laboratory analyses

Total RNA was extracted from SAT, PAT, and EAT by processing samples through the RNeasy Lipid Tissue Mini Kit (Qiagen, GmbH, Germany), following the manufacturer’s instructions. RNA concentration and purity was determined using a NanoDropTM 1,000 Spectroscophotometer (SaveenWerner, Sweden), observing a mean concentration of 29.3 ng/mL and a purity of 1.7 (absorbance ratio at 260 and 280 nm). cDNA was then retro-transcribed starting from 5 ng/mL of RNA for each sample and using the qScript™ cDNA superMix commercial kit (Quanta Biosciences, United States). Gene expression analyses were measured with TaqMan® assays (Applied Biosystems, CA, United States), as follows: L-Galectin 9 (Hs00247135_m1), CD206 (Hs00267207_m1) and NOS2 (Hs01075529_m1). Real-Time qPCR was performed using the TaqMan® Universal PCR Master Mix (cat. n. 4,324,018) on a ViiATM7 instrument (Applied Biosystems, CA, United States). The ΔΔCt method was applied to determine the mRNA levels in each reaction, using the β2-Microglobulin (Hs99999907_m1) as the normalizer internal gene, and expressed as relative quantification (RQ) to a reference sample (28). Gene expression of CD163, CD68, CD3, and CD31, representing macrophages, T cells and endothelial cells, respectively, was analyzed through RT-PCR to determine the different cell types present in each AT sample, as previously described (27).

Whole blood from each patient was centrifuged at 2,500 g for 10` and the isolated serum was conserved at −80°C until use. Commercially available enzyme-linked immunosorbent assays (ELISA) were used to determine the circulating levels of L-Gal 9 (R&D Systems, NE, US), CD206 (RayBotech, GA, United States) and NOS2 (LifeSpan BioSciences, WA, United States), following the manufacturer’s instructions. All samples were analyzed as duplicates. The intra-assay coefficient of variation (CV) in our laboratory were 6.7, 3.5, and 5.8%, respectively. Routine patient’s analyses were performed by conventional laboratory methods.

### 2.3. Statistical analyses

The characteristics of patients are reported as numbers and percentages or as median values and 25th and 75th percentiles. The vast majority of the variables were skewed distributed, therefore non-parametric analyses were performed, including the Mann–Whitney U-test to compare the two groups, the Friedmans’ test coupled to the Wilcoxon signed-rank test to compare gene expression in the individual AT compartments. The Spearmann’s rho was used for correlations and trend lines applied in figures when being statistically significant. Furthermore, the Chi-square test was used for differences in categorical variables between groups. A value of *p* < 0.05 was considered statistically significant, and Bonferroni correction for multiple comparisons was applied as specified. Statistical analyses were performed using SPSS version 28 (SPSS Inc., IL, United States).

## 3. Results

### 3.1. Patients characteristics

Clinical and laboratory parameters of the 74 recruited subjects, 52 CHD and 22 CTRLs are reported in Table 1. The proportion of men was higher in the CHD group, as well as glomerular filtration rate (GFR), HbA1c levels and the use of aspirin, beta-blockers, statins and other lipid-lowering drugs. The latter are possibly responsible for the lower total-and LDL-cholesterol levels found in CHD patients compared to CTRLs.

**Table 1:** Characteristics of the patient populations.

	CTRLs (*n* = 22)	CHD (*n* = 52)	*p* value
Age (years)	69 (63, 71.5)	66.5 (62,71.8)	
Male (%)	**11 (50%)**	**40 (76.92%)**	**0.04**
Smoker (previous/current)	10 (45.45%)	31 (59.6%)	
Weight (Kg)	82.5 (77.5, 107.0)	85 (70.3, 95.5)	
Height (m)	1.75 (1.7, 1.8)	1.77 (1.7, 1.8)	
Waist (cm)	90 (88, 101)	92 (86, 98)	
BMI (kg/m^2^)	28.4 (24.6, 31.6)	27.3 (23.8, 30.1)	
SBP (mmhg)	140 (115, 163)	140 (125, 160)	
DBP (mmhg)	79 (70, 86)	80 (70, 87)	
**Cardiovascular status**			
Previous AMI (%)	**2 (9.1%)**	**20 (38.5%)**	**0.025**
Angina (%)	**0 (0%)**	**24 (46.2%)**	**<0.001**
PCI (%)	**0 (0%)**	**20 (38.5%)**	**0.002**
Hypertension (%)	9 (40.9%)	28 (53.8%)	
Diabetes type I and II (%)	3 (13.6%)	14 (26.9%)	
Heart failure (%)	1 (4.5%)	3 (5.8%)	
**Medications**			
Aspirin (%)	**9 (40.9%)**	**45 (86.5%)**	**<0.001**
Other antiplatelet (%)	0 (0%)	14 (26.9%)	
ACEi/ATII (%)	11 (50%)	24 (46.15%)	
Beta-blockers (%)	**6 (27.3%)**	**32 (61.5%)**	**0.015**
Statins (%)	11 (50%)	37 (71.2%)	
Lipid-lowering agents (%)	1 (4.54%)	10 (19.2%)	
NSAIDs (%)	0 (0%)	2 (3.9%)	
Insulin (%)	0 (0%)	6 (11.5%)	
Anti-diabetic drugs (%)	3 (13.6%)	11 (21.2%)	
Diuretics (%)	5 (22.7%)	7 (13.46%)	
**Laboratory values**			
hsCRP (mg/L)	1.00 (1.0, 2.0)	0.91 (0.49, 1.77)	
Troponin T (ng/L)	11.5 (9, 25)	13 (9, 22)	
TC (mmol/L)	**3.9 (2.8, 4.6)**	**3.1 (2.7, 3.4)**	**0.026**
HDL-C (mmol/L)	1.10 (0.9, 1.3)	0.97 (0.8, 1.1)	
LDL-C (mmol/L)	**2.18 (1.8, 2.9)**	**1.83 (1.4, 2.2)**	**0.02**
Triglycerides (mmol/L)	1.02 (0.9, 1.7)	1.22 (1.0, 1.8)	
Glucose (mmol/L)	5.6 (4.9, 6.3)	5.6 (4.9, 6.6)	
HbA1c (mmol/mol)	**36 (33, 38)**	**39 (36, 51)**	**0.011**
GFR (%)	**80 (68, 91)**	**90 (75, 95)**	**0.03**
Carbamide (mmol/L)	6.2 (5.1, 7.3)	5.5 (4.7, 6.7)	
Creatinine (μmol/L)	80 (64.8, 91)	76 (67, 85)	
Uric acid (μmol/L)	317 (258, 396)	313 (271, 362)	

Median (25, 75 percentiles) or number proportions are reported.

Chi square test was used for differences between groups in categorical variables, while the Mann–Whitney *U*-test was used for continuous variables. A *p* value <0.05 was considered statistically significant, marked in bold.

BMI, Body Mass Index; SBP, Systolic Blood Pressure; DBP, Diastolic Blood Pressure; AMI, Acute Myocardial Infarction; PCI, Percutaneous Coronary Intervention; ACEi, Angiotensin-Converting Enzyme inhibitors; ATII, Angiotensin II receptor antagonist; NSAIDs, non-steroidal anti-inflammatory drugs; hsCRP, high sensitive C-reactive protein; TC, total cholesterol; HDL-C, high-density lipoprotein cholesterol; LDL-C, low-density lipoprotein cholesterol; HbA1C, glycosylated hemoglobin A1c; GFR, Glomerular filtration rate.

### 3.2. Gene expression and circulating proteins in CHD and CTRLs

L-Gal 9, CD206, and NOS2 gene expression in the three AT compartments was overall similar in CHD patients and CTRLs (Table 2). Circulating L-Gal 9 and CD206 levels were similar in the two populations, while NOS2 circulating levels were significantly higher in CHD patients as compared to CTRLs (*p* = 0.012).

**Table 2:** AT expression and serum concentration of macrophage phenotype regulating parameters in CHD and control patients.

		CTRLs	CHD	*p* value
**AT L-Gal9**				
	SAT	0.63 (0.4, 1.18)	0.86 (0.47, 1.25)	0.52
	PAT	0.70 (0.47, 1.45)	1.08 (0.55, 1.47)	0.23
	EAT	0.70 (0.56, 0.97)	0.92 (0.48, 1.26)	0.46
**AT CD206**				
	SAT	0.62 (0.32, 1.00)	0.57 (0.36, 0.78)	0.80
	PAT	0.73 (0.31, 1.11)	0.72 (0.37, 1.11)	0.61
	EAT	0.54 (0.43, 0.95)	0.57 (0.34, 0.81)	0.58
**AT NOS2**				
	SAT	1.13 (0.78, 2.20)	0.9 (0.38, 1.74)	0.29
	PAT	0.59 (0.43, 1.11)	0.67 (0.34, 1.26)	0.93
	EAT	0.49 (0.37, 0.74)	0.52 (0.31, 0.90)	0.70
*Circulating levels*				
**L-Gal 9**	ng/mL	6.21 (5.28, 7.72)	5.88 (5.01, 7.56)	0.98
**CD206**	ng/mL	312.04 (234.68, 474.86)	374.11 (228.71, 895.11)	0.24
**NOS2**	pg/mL	**380.59 (254.11, 497.51)**	**573.05 (383.21, 935.67)**	**0.01**

AT expression is reported as relative quantification (RQ) values, while circulating levels are expressed as serum concentration. Median (25, 75 percentiles) values are reported. The Mann–Whitney *U*-test was used to compare gene expression between CTRLs and CHD patients; a *p* < 0.05 was considered statistically significant. NOS2 circulating levels were significantly higher in CHD patients compared to CTRLs (*p* < 0.01; marked in bold).

For abbreviations: see text.

### 3.3. Expression of the selected genes in the AT compartments

**Figure 1:**
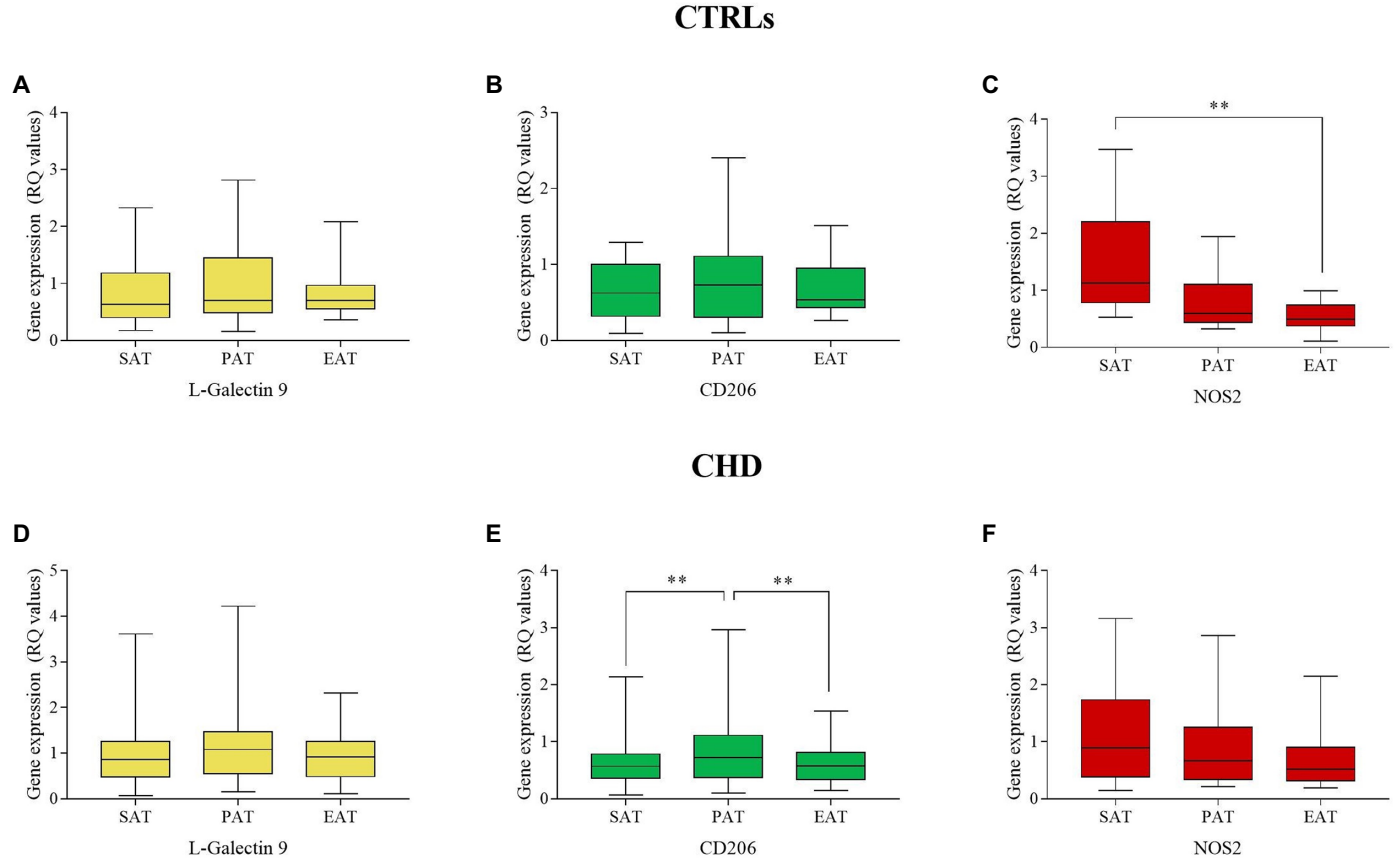
Gene expression of L-Gal 9, CD206, and NOS2 in SAT, PAT, and EAT compartments. Data are reported as RQ values and box plots indicate the median values, 25th and 75th percentiles, while error bars report 10th and 90th percentiles. Gene expression in AT compartments was evaluated through the Friedman’s test coupled to the Wilcoxon signed-rank test and a value of *p* < 0.05 was considered statistically significant. L-Gal9 expression was similar in all ATs in CTRLs **(A)** and CHDs **(D)**; CD206 was similarly expressed in CTRLs **(B)**, but significantly lower in SAT and EAT as compared to PAT in CHDs **(E)**; NOS2 expression was significantly lower in EAT vs. SAT in CTRLs **(C)**, but similarly expressed among ATs in CHDs (F)**p < 0.01.

In CTRLs, L-Gal 9, and CD206 were similarly expressed in the three AT compartments (Figures 1A,B), whereas NOS2 showed the lowest expression in EAT (Figure 1C), in which values were significantly lower than those relative to SAT (*p* = 0.007). In CHD patients, no significant differences were found for L-Gal 9 and NOS2 expression (Figures 1D,F), although EAT showed the lowest NOS2 values. CD206 expression was significantly lower in SAT and EAT as compared to PAT (*p* = 0.003 and *p* = 0.006, respectively; Figure 1E).

### 3.4. Correlations in gene expression of L-gal 9, CD206, and NOS2 between the different AT compartments

**Figure 2:**
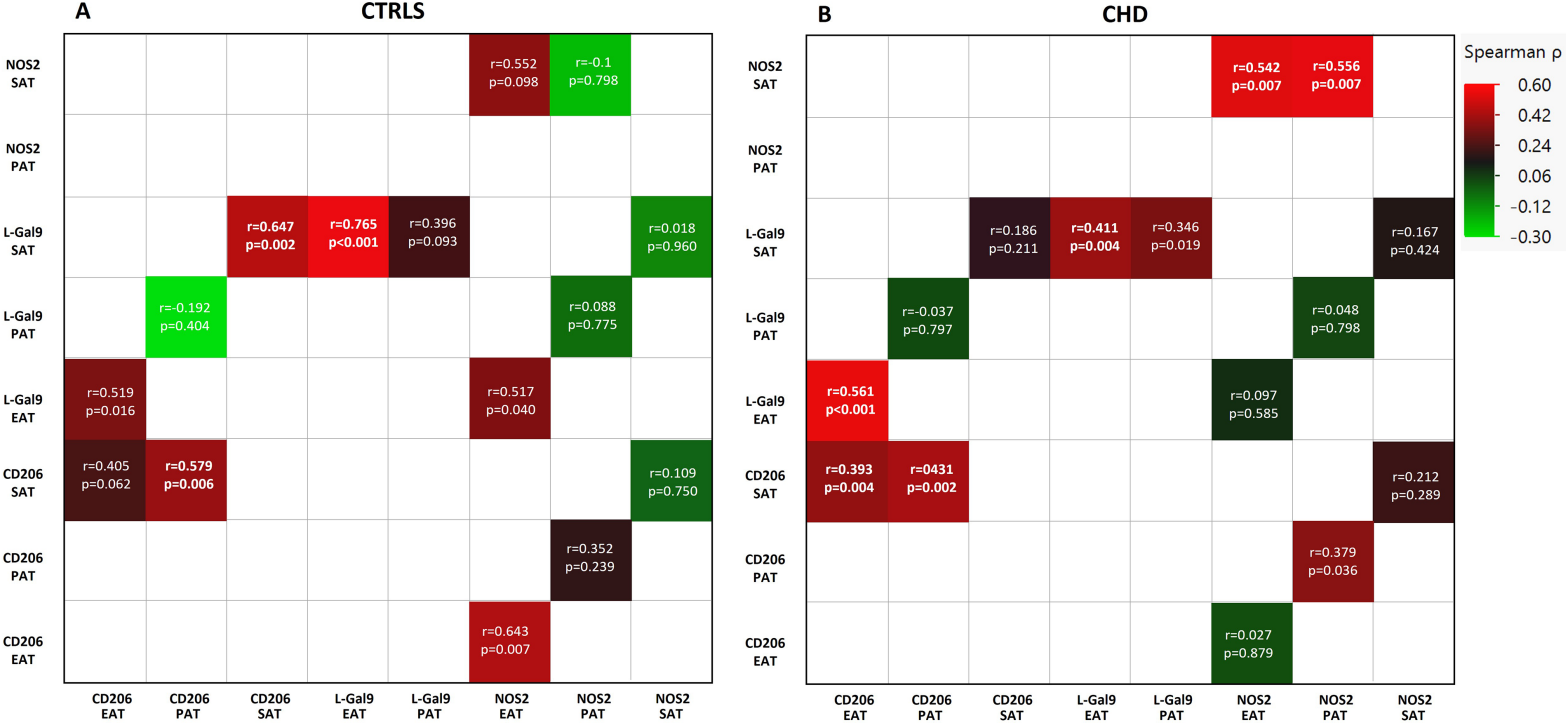
Correlations between the expression of L-Gal9, CD206, and NOS2 in SAT, PAT, and EAT compartments in CTRLs **(A)** and CHD patients **(B)**. Correlations are reported as heatmap following Spearman ρ analyses, and those still significant after Bonferroni correction are marked in bold.

In the CTRL group, L-Gal 9 expression in SAT positively correlated to that in EAT (*r* = 0.765, *p* < 0.001), while CD206 expression in SAT correlated to its expression in PAT (*r* = 0.579, *p* = 0.006) (Figure 2A). In CHD patients, L-Gal 9, CD206, and NOS2 expression in SAT positively correlated with their expression in EAT (*r* = 0.411, *p* = 0.004; *r* = 0.393, p = 0.004 and *r* = 0.542, *p* = 0.007, respectively) (Figure 2B; Supplementary Figures 1A,C,E). In addition, CD206 and NOS2 expression in SAT positively correlated with their expression in PAT (*r* = 0.431, *p* = 0.002 and *r* = 0.556, *p* = 0.007, respectively) (Figure 2B; Supplementary Figures 1B,D).

No significant correlations were found between circulating L-Gal 9, CD206, and NOS2 levels and their corresponding gene expression in SAT, PAT, and EAT in neither CTRL nor CHD patients (Supplementary Table 1).

### 3.5. Correlations between expression of L-gal 9, CD206, and NOS2 within each AT compartment

When analyzing for the inter-relationships between gene expression of the actual markers within each AT, significant correlations (Bonferroni’s corrected; 18 comparisons, *p* < 0.002) were found between L-Gal 9 and CD206 within SAT (*r* = 0.647, *p* = 0.002) in CTRLs (Figure 2A; Supplementary Figure 2A) and within EAT (*r* = 0.561, *p* < 0.001) in CHD (Figure 2B; Supplementary Figures 2B).

### 3.6. Correlations between L-gal 9, CD206, and NOS2 expression and cell markers in the AT compartments in CHD patients

**Figure 3:**
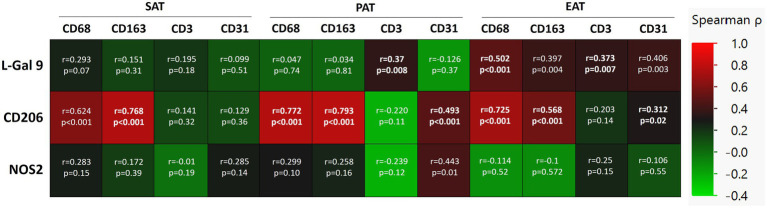
Correlations between L-Gal 9, CD206, and NOS2 expression and the cell markers CD68, CD163, CD3, and CD31 in SAT, PAT, and EAT in CHD patients. Correlations are reported as heatmap following Spearman ρ analyses, and those still significant after Bonferroni correction (36 comparisons; *p* < 0.0014) are marked in bold.

CD163 and CD68, CD3, and CD31, the most representative markers of macrophages, T cells and endothelial cells, respectively, were analyzed in SAT, PAT, and EAT of CHD patients to detect the specific cellular subtypes in each AT compartment. The previously analyzed markers were detectable in the three AT compartments, with an overall similar distribution (27). When matching these data with L-Gal 9, CD206, and NOS2, we found that L-Gal 9 expression in PAT and EAT positively correlated to CD3 expression (*r* = 0.37, *p* = 0.008 and *r* = 0.373, *p* = 0.007, respectively; although no longer statistically significant after Bonferroni’s correction; Figure 3). After testing for multiple corrections, L-Gal 9 expression correlated still significantly to CD68 in EAT (*r* = 0.502, *p* < 0.0001; Figure 3), while CD206 expression correlated positively to CD163 and CD68 in all AT compartments (*p* < 0.0001, all; Figure 3) and with CD31 in PAT (*r* = 0.493, *p* < 0.0001, Figure 3).

### 3.7. L-gal 9, CD206, and NOS2 AT expression according to lipid profile and anthropometric characteristics in CHD patients

**Figure 4:**
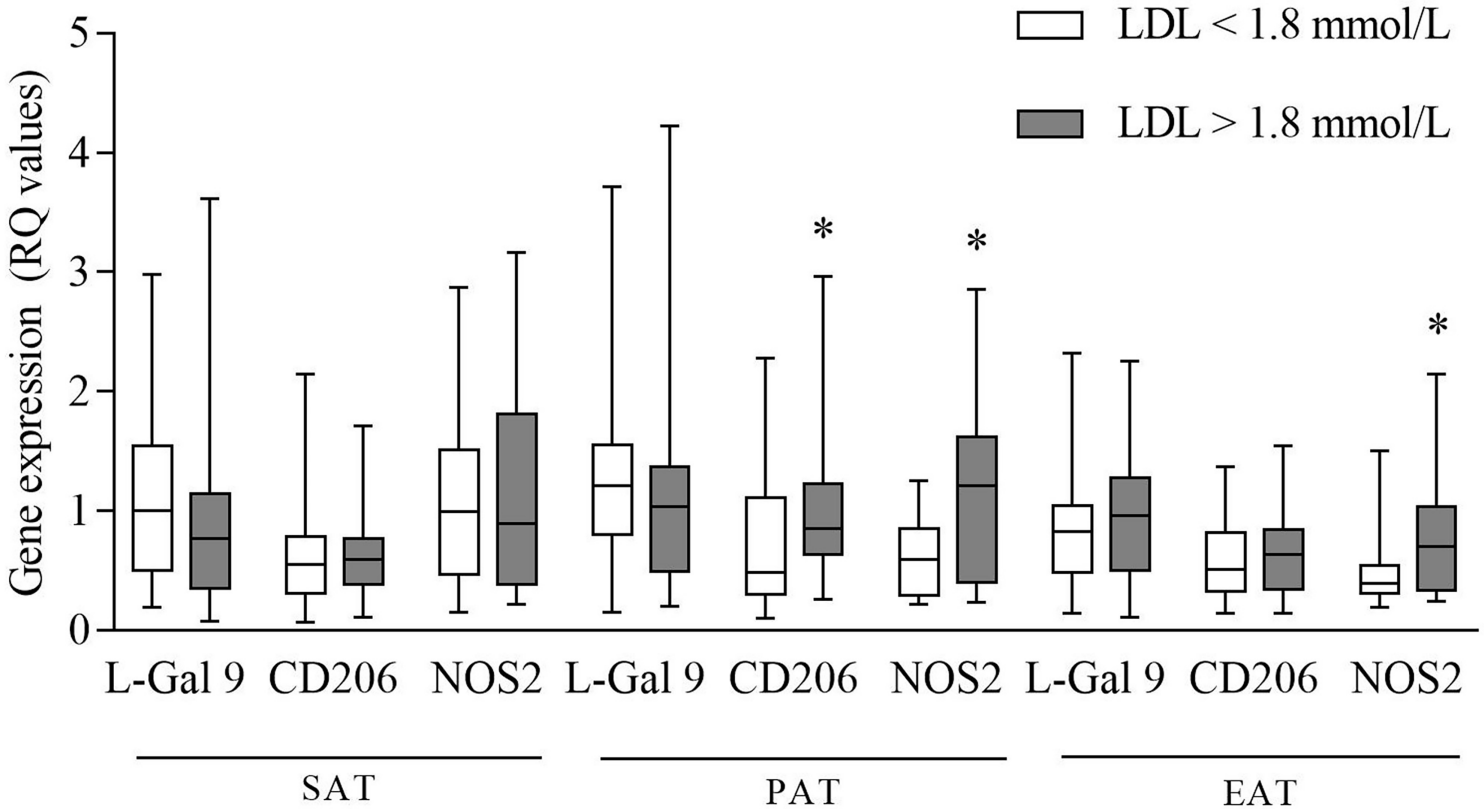
Gene expression of L-Gal 9, CD206, and NOS2 in CHD patients with LDL-C levels below and above the median value (1.8 mmol/L). Data are reported as RQ values and box plots indicate the median values, 25th and 75th percentiles, while error bars report 10th and 90th percentiles. Mann–Whitney *U*-test was applied and a value of *p* < 0.05 was considered statistically significant. Above median vs. below median: PAT: higher expression of CD206 and NOS2. In EAT: higher expression of NOS2. *p < 0.05.

Higher NOS2 expression was found in both PAT and EAT in CHD subjects with LDL-C levels above compared to those with levels below the median value of 1.8 mmol/L (Figure 4). Also, CD206 expression in PAT was higher in patients with LDL-C above median (Figure 4).

**Figure 5:**
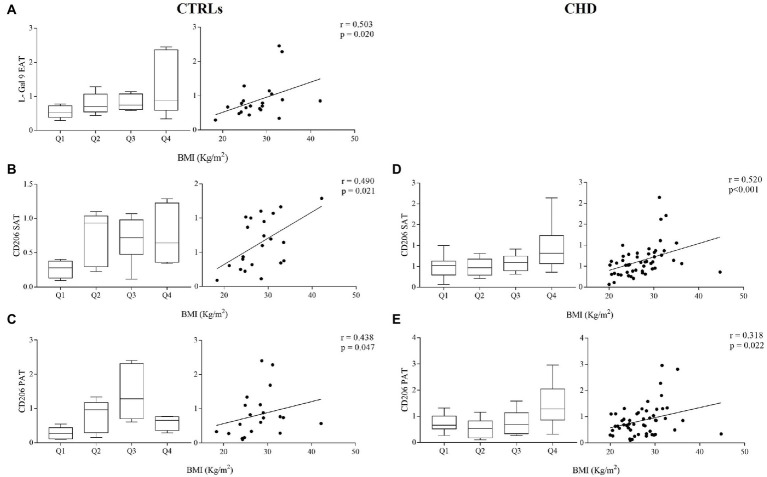
Gene expression of L-Gal 9 in CTRLs **(A)** and CD206 in CTRLs **(B,C)** and CHD patients **(D,E)** either stratified based on subject’s BMI quartiles (left graph) or as correlation with continuous BMI values (right graph). Data are reported as RQ values and box plots indicate the median values, 25th and 75th percentiles, while error bars report 10th and 90th percentiles. Spearman ρ analyses were performed and only significant correlations after Bonferroni’s correction (27 comparisons; *p* < 0.0018) are reported.

Next, we investigated the association between L-Gal 9, CD206, and NOS2 gene expression in the different compartments and the most relevant anthropometric parameters, also dividing gene expression data in quartiles to show potential cut-off values. We observed in CTRLs only a significant positive correlations between EAT L-Gal 9 expression and BMI (Figure 5A), weight and waist (Supplementary Table 2). In both CTRLs and CHD patients, significant correlations were found between CD206 gene expression in SAT and PAT and subjects’ BMI (Figures 5B–E), although the correlation in PAT appeared less strong and not statistically significant after Bonferroni’s correction, as also did the correlation between CD206 gene expression in SAT and PAT in CTRLs.

### 3.8. Correlations between circulating levels of L-gal 9, CD206, NOS2, and serum lipids and CRP

**Figure 6:**
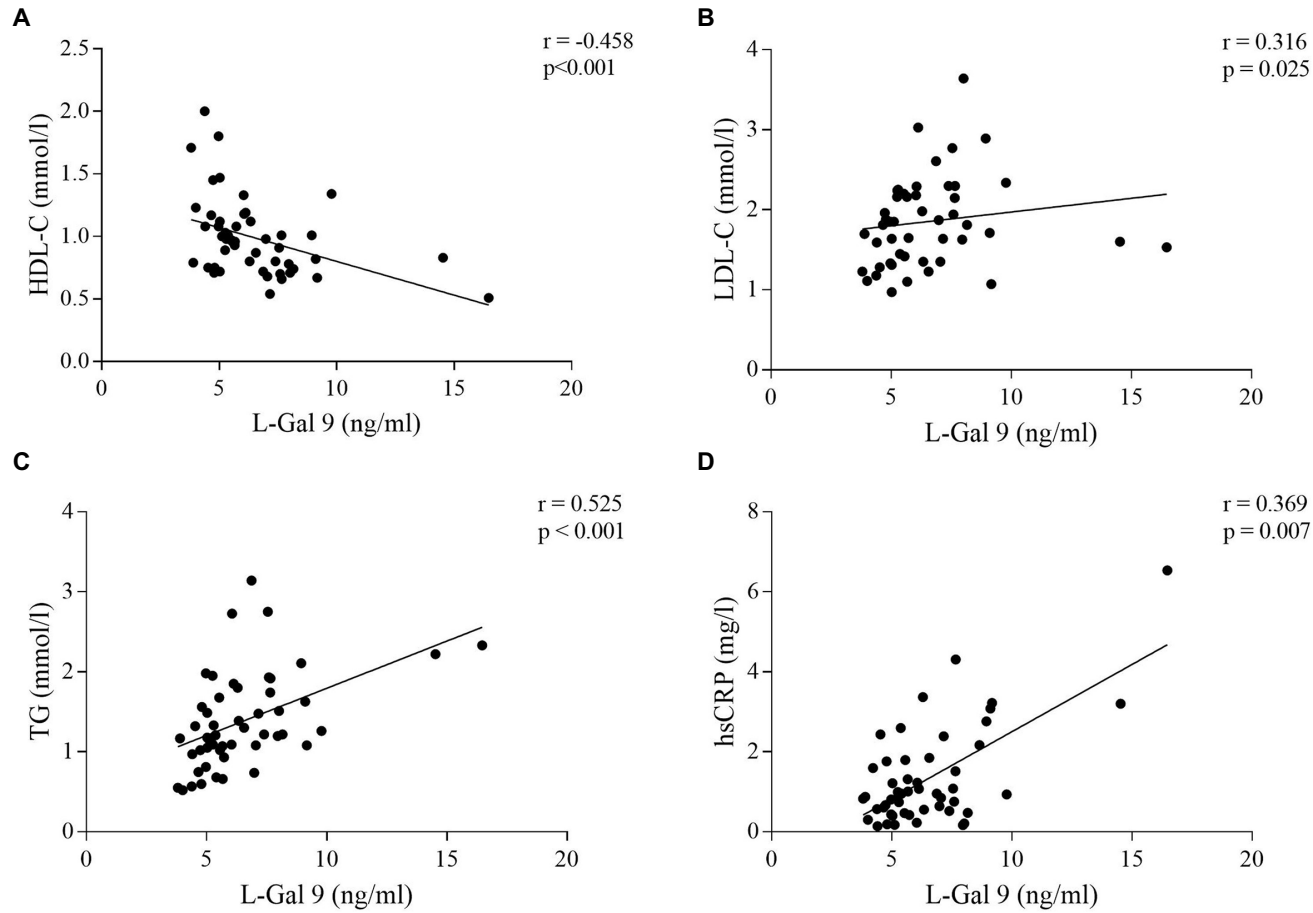
Correlations between L-Gal 9 circulating levels and HDL-C **(A)**, LDL-C **(B)**, TG **(C)**, and hsCRP **(D)** in CHD patients. Spearman ρ analyses were performed and Bonferroni’s correction was applied (6 comparisons; *p* < 0.008).

No significant correlations were found between circulating levels of L-Gal 9, CD206 and NOS2 and serum lipids and CRP in the CTRL group. In CHD patients, L-Gal 9 circulating levels correlated inversely with HDL-C (*r* = −0.458, *p* < 0.001; Figure 6A) and positively with LDL-C (*r* = 0.316, *p* = 0.025; Figure 6B), TG (*r* = 0.525, *p* < 0.001; Figure 6C) and hsCRP (*r* = 0.369, *p* = 0.007; Figure 6D). However, after Bonferroni’s correction, the correlation to LDL-C lost its statistical significance (Supplementary Table 3). CD206 and NOS2 circulating levels were not correlated to any serum lipid and to hsCRP, and none of the circulating markers correlated significantly to anthropometric measures (Supplementary Table 3).

## 4. Discussion

In this clinical case–control study we aimed to explore expression of genes linked to the polarization status of macrophages in SAT, PAT, and EAT in CHD patients undergoing coronary artery bypass grafting and in controls undergoing aortic valve replacement.

Our main findings were that expression of the selected genes, L-Gal 9, CD206 and NOS2, that to some degree reflects the macrophage polarization process, (I) did not differ between CHD and control patients; (II) showed a few differences between the various AT compartments: NOS2 was least expressed in control subjects EAT, whereas CD206 was most expressed in PAT of CHD patients; (III) associated with the presence of macrophages in all compartments, except for NOS2; (IV) in specific cases associated with LDL-C levels and modestly with BMI; (V) did not associate with circulating levels of the markers.

The lack of differences in AT gene expression between CHD and CTRLs might be explained considering that the control cohort was composed of patients undergoing aortic valve replacement, thus presenting a cardiopathy. In fact, very recently it was reported that such patients possess pro-inflammatory activation of ascending aortic AT and EAT (29). Another explanation might be that serum total cholesterol and LDL-C were higher in CTRLs compared to CHD patients, and some CTRLs had hypertension and diabetes mellitus. Moreover, a large part of the recruited CHD patients were under treatment with aspirin and statins, both known to exert an anti-inflammatory activity (30, 31), thus possibly further attenuating potential differences.

When comparing gene expression levels of the investigated molecules in the different AT compartments, we did not find differences in L-Gal 9 expression between SAT, PAT and EAT in either CTRLs or CHD patients. Several *in vitro* studies have demonstrated that L-Gal 9 is involved in the modulation of a variety of biological processes, including cell aggregation and adhesion, regulation of T cell pools, as well as the modulation of macrophages polarization (23, 24). Only a few studies have evaluated L-Gal 9 expression in AT, and demonstrated that it is predominantly expressed in the stromal vascular fraction (SVF), composed of various cell types. It is possible that, overall, L-Gal 9 expression in the various AT compartments is similarly and tightly controlled. We observed lower CD206 expression in SAT and EAT compared to PAT in both groups, statistically significant in CHD patients only, possibly due to the lower number of CTRLs. This is in line with the different origin and vascularization of PAT (2, 7). Lower CD206 expression in EAT and SAT might be important because in concomitance with atherosclerosis progression the number of M2 macrophages in the plaques decreases (32) in various tissues including AT, with consequent formation of crown-like structures, a typical hallmark of chronic fat tissue inflammation that possibly hints CHD (33). We found NOS2 to be less expressed in EAT with respect to the other compartments, although statistical significance was reached only compared to SAT in the control group. Very recently, it was reported that adipocytes in EAT generated exosomes containing NOS2 (34). Thus, it is possible that the NOS2 we measured was adipocyte-derived, rather than of macrophage origin. Accordingly, its expression did not correlate with any macrophage, T cell or endothelial cell marker. The lower NOS2 expression in EAT in controls points to a specific, stricter regulation of this molecule in EAT, which might be lost in CHD (**Graphical abstract,** point **a**). Moreover, the EAT and SAT expression of NOS2 was positively associated only in the CHD group, in which also L-Gal 9 and CD206 were positively inter-correlated in EAT, suggesting activation of a compensatory anti-inflammatory mechanism specifically occurring in EAT in presence of CHD (**Graphical abstract,** point **b**).

In CHD patients, specifically in EAT, L-Gal 9 expression positively and strongly correlated to CD68 expression, a cellular marker identifying macrophages (35), thus the L-Gal 9 findings described above seems to be mainly related to its macrophage expression. As a consequence, the positive association observed specifically in EAT between L-Gal 9 and CD206 expression in CHD patients might confirm our hypothesis of a L-Gal 9 signaling toward an anti-inflammatory M2 profile in CHD patients, to compensate a local cardiac AT low-grade inflammation (36) (**Graphical abstract**, point **b**). Furthermore, specifically in PAT and EAT, a positive, albeit milder association was observed between L-Gal 9 and the expression of T cell receptor CD3. This is particularly of interest as several studies have demonstrated that L-Gal 9 acts also by binding and promoting the activity of death receptor 3, involved in the expansion of regulatory T cells number and activity (37), known to exert a fundamental role in AT homeostasis (38). CD206 gene expression was strongly associated with that of CD163 and CD68 in all AT analyzed. This could be expected as both CD206 and CD163 are commonly expressed by AT resident macrophages, mostly characterized by an M2-like signature (39, 40), while CD68 is commonly used as a macrophage-specific cell marker (35), regardless of the cell phenotype. Interestingly, specifically in PAT, we observed a positive association between CD31 expression, representing endothelial cells, and CD206. Notably, it has previously been demonstrated in humans that the vascular density and amount of endothelial cells in visceral AT is higher as compared to SAT, with a higher angiogenic and inflammatory profile (41). Furthermore, a pre-clinical model of infarcted rats showed that the administration of stem cells isolated from PAT promoted myogenic differentiation, with consequent efficient cardiac repair (42). Hence, the positive association that we observed between CD206 and CD31 expression selectively in PAT in CHD patients may be due to a possible reparatory mechanism.

As for circulating concentration of the investigated molecules, no difference in L-Gal 9 levels between CHD and controls was found. Increased levels of L-Gal 9 have been described in a wide range of pathologic conditions, like autoimmune and infectious diseases and in diabetes mellitus (43–45). Conversely, reduced levels were reported in patients with acute coronary syndrome as compared to patients without CAD (43, 44). We found L-Gal 9 circulating levels to be positively associated with LDL-C, TG, and hsCRP concentrations, and inversely correlated to HDL-C, only in CHD patients. As previously hypothesized, L-Gal 9 may increase as a compensatory response to the inflammatory environment in CHD subjects. We found no differences in CD206 circulating levels between CHD and control patients. To our knowledge, no study has to date been reported on CD206 serum concentrations in subjects with CVDs. In addition, no correlations were found between serum CD206 concentration and serologic and clinical parameters, neither in CHD nor in CTRLs subjects. Circulating levels of NOS2 were significantly higher in CHD patients vs. CTRLs. NOS2, also known as inducible Nitric Oxide Synthase (iNOS), is a key enzyme synthesizing nitric oxide (NO) under specific inflammatory conditions, including atherosclerosis (46, 47). Indeed, while small amounts of NO produced by eNOS are known to exhibit atheroprotective effects (48), enhanced NO production upon NOS2 activation leads to cytotoxicity and oxidative stress (49), thus potentially contributing to CVD development. Recent studies revealed that NOS2 can be found in circulating macrovesicles in its inactive form, while activated when macrovesicles are internalized into target cells (50). As such, septic intensive care unit patients displayed a strong increase in NOS2 circulating levels as compared to non-septic, followed by decreased levels upon effective therapy (50). Within this picture, the observed slight increase of NOS2 concentration in CHD patients might be compatible with low-grade inflammation (**Graphical abstract**, point **a**). NOS2 serum levels did not correlate to any serologic or clinical parameter. We could not show any significant correlation between circulating levels of L-Gal 9, CD206 and NOS2 and their corresponding gene expression in the three AT compartments, suggesting that tissue-specific events may not be reflected in circulatory levels, as previously reported (12).

We observed a modest association between L-Gal 9, CD206 and NOS2 with BMI. SAT expression of CD206 associated significantly with BMI and weight in CHD patients, but not in CTRLs, probably due to the lower number in this group. On the other hand, the positive association found specifically in CTRLs between EAT L-Gal 9 expressions and BMI, weight and waist circumference might be a physiologic compensatory anti-inflammatory mechanisms in response to increased amount of AT. This mechanisms might be lost in CHD patients, possibly contributing to the establishment of a pro-inflammatory environment in this specific AT compartment. The relationship between AT macrophage polarization and anthropometric measures has not been fully investigated yet. It has been reported that pro-inflammatory SAT macrophages were increased with BMI, in parallel with a decrease in their anti-inflammatory counterpart; however this was not found in subjects with BMI <30 kg/m^2^ and in VAT (51), thus highlighting that this expression pattern specifically occurs in obese SAT.

To our knowledge, very few studies have investigated the influence of circulating cholesterol levels on AT inflammation. The administration of a diet rich in saturated fatty acids in mice led to a significant increase in LDL-C and AT expression of CD206 and CD11c, the latter used to identify M1 macrophages, together with the activation of nuclear factor-kappa B (NF-kB), thus suggesting a general increased AT inflammation (52). However, to date, no human studies have been reported. Interestingly, we observed that specifically in CHD patients with LDL-C levels above the median (1.8 mmol/L), PAT and EAT NOS2 expressions were significantly increased as compared to subjects with LDL-C levels below median. These observations suggest that CHD subjects with high LDL-C may be characterized by an increased inflammatory environment occurring selectively in cardiac ATs (**Graphical abstract**, point **c**).

There are several limitations in our study, mainly related to the impossibility to exclude any degree of subclinical atherosclerosis in the control group, as discussed. Moreover, we cannot fully rule out a potential AT dysfunction in our recruited controls. Finding an appropriate control group for this kind of investigation is challenging. Cardiac surgery indications are a mandatory prerequisite for cardiac AT biopsy collection, which necessarily are based on some sort of cardiac disease, potentially impacting on, or associated with AT dysfunction. Also, a high proportion of CHD patients were treated with aspirin and statins (30, 31), which possibly impacts macrophage polarization. Furthermore, we do not have any imaging information, such as CT scans, to measure the AT volume in the various cardiac and subcutaneous locations, and we have made a selection of polarization markers. Finally, being an observational study, only the associations and not the causality could be investigated. On the other side, the strength of this experimental design is the concomitant availability of the two compartments of cardiac AT in addition to the pre-sternal subcutaneous AT, thus allowing us to separately study the macrophage polarization at a molecular level in the different AT compartments.

In conclusion, through the analysis of macrophage polarization markers in pericardial, epicardial and subcutaneous AT, this study suggests that CHD patients might be characterized by an increased low-grade inflammation specifically occurring in EAT. A compensatory anti-inflammatory mechanism involving the L-Gal 9-CD206 axis might be a possible consequence, as the tight regulation of pro-inflammatory NOS2 signaling observed in the controls may partly be lost in CHD patients. Hence, cardiac, and especially EAT macrophage polarization might be considered a promising field of investigation to target more precisely the inflammatory status in cardiovascular disease.

## Data availability statement

The original contributions presented in the study are included in the article/Supplementary material, further inquiries can be directed to the corresponding author.

## Ethics statement

The studies involving human participants were reviewed and approved by Regional Ethics Committee of North Norway (#2016/411). The patients/participants provided their written informed consent to participate in this study.

## Author contributions

BP carried out the experiments, acquired the data, and wrote the first draft of the manuscript and performed the statistical analyses. TT, BB, SÅ, and CH recruited the subjects, performed the assessments of patients, and critically reviewed the manuscript for intellectual content. IS, TO, HA, SS, and NR conceived and designed the study and handled the founding. IS, TO, and NR handled supervision and critically reviewed the manuscript for intellectual content. All the authors read and approved the final manuscript.

## Funding

We thank the Italian Society of Pharmacology (Italy) for the scholarship to BP and the Stein Erik Hagens Foundation for Clinical Heart Research, Oslo (Norway) for financial support. The funder had no role in the design of the study, in the collection, analyses, or interpretation of data, in the writing of the manuscript or in the decision to publish the results.

## Conflict of interest

The authors declare that the research was conducted in the absence of any commercial or financial relationships that could be construed as a potential conflict of interest.

## Publisher’s note

All claims expressed in this article are solely those of the authors and do not necessarily represent those of their affiliated organizations, or those of the publisher, the editors and the reviewers. Any product that may be evaluated in this article, or claim that may be made by its manufacturer, is not guaranteed or endorsed by the publisher.
