# Supplementary figures and images for: Macrophage polarization markers in subcutaneous, pericardial, and epicardial adipose tissue are altered in patients with coronary heart disease
Source: Front Cardiovasc Med. 2023 Mar 2;10:1055069. doi: 10.3389/fcvm.2023.1055069 (PMC10017535; doi:10.3389/fcvm.2023.1055069)

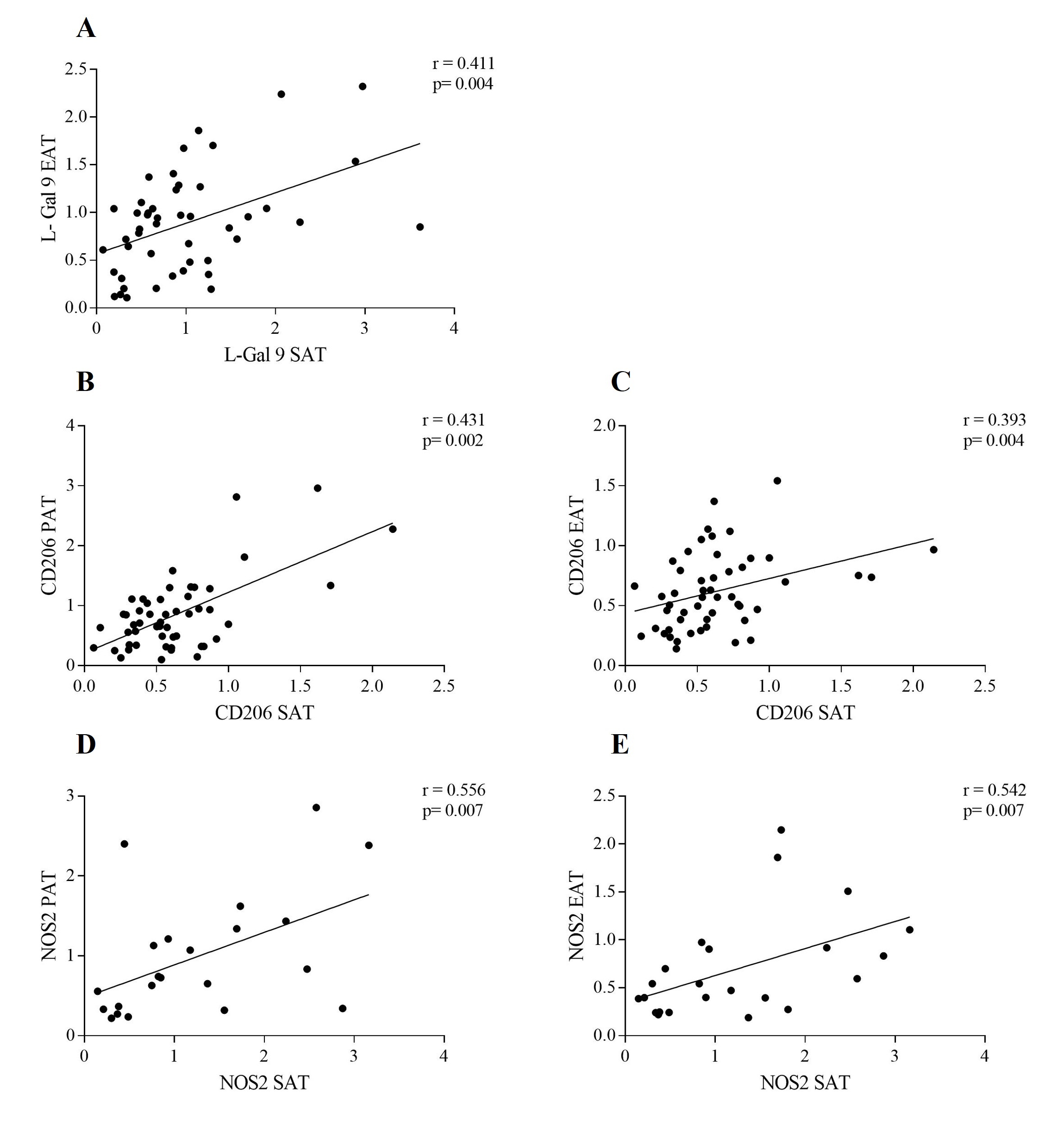

Supplement: Supplementary file 1 [file Image_1.JPEG]

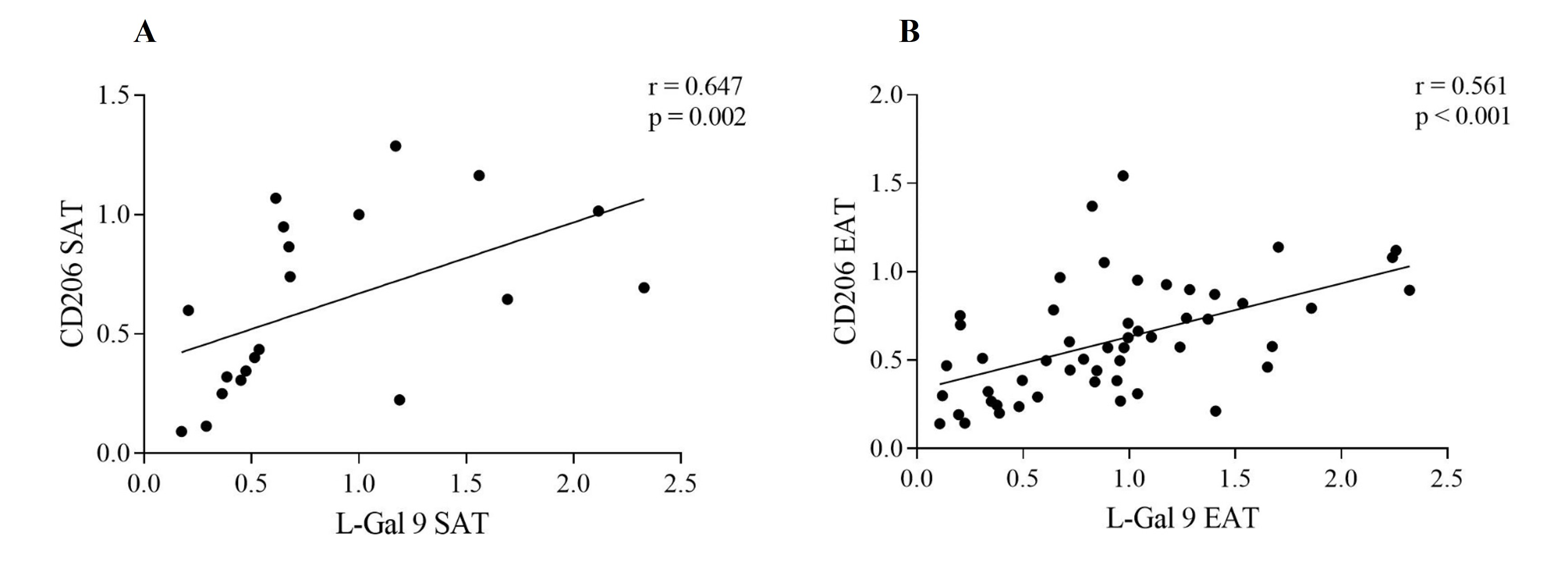

Supplement: Supplementary file 2 [file Image_2.JPEG]
