# Supplementary material for: Macrophage polarization markers in subcutaneous, pericardial, and epicardial adipose tissue are altered in patients with coronary heart disease
Source: Front Cardiovasc Med. 2023 Mar 2;10:1055069. doi: 10.3389/fcvm.2023.1055069 (PMC10017535; doi:10.3389/fcvm.2023.1055069)
Supplement: Supplementary file 3 [file Table_1.docx]

Supplementary Material

# Supplementary Tables

**Supplementary Table 1.** *Correlations between circulating levels of L-Gal 9, CD206 and NOS2 and their corresponding gene expression in AT compartments in CTRLs and CHD patients*.

| **CTRLs** | | | | | **CHD** | | | | |
| --- | --- | --- | --- | --- | --- | --- | --- | --- | --- |
|  |  | **SAT** | **PAT** | **EAT** |  |  | **SAT** | **PAT** | **EAT** |
| **L-Gal 9** ng/ml | r  p | 0.122  0.609 | -0.079  0.733 | 0.104  0.654 | **L-Gal 9** ng/ml | r  p | 0.058  0.749 | -0.032  0.823 | -0.057  0.69 |
| **CD206**  ng/ml | r  p | -0.029  0.911 | -0.418  0.107 | -0.324  0.205 | **CD206** ng/ml | r  p | -0.2  0.198 | -0.084  0.59 | 0.093  0.548 |
| **NOS2**  pg/ml | r  p | -0.37  0.293 | 0.042  0.907 | -0.413  0.183 | **NOS2**  pg/ml | r  p | -0.082  0.703 | -0.253  0.203 | 0.23  0.212 |

**Supplementary Table 2.** *Correlations between CHD L-Gal 9, CD206 and NOS2 gene expression in SAT, PAT and EAT and the most relevant anthropometric parameters*. Significant correlations after Bonferroni`s correction (27 comparisons; p < 0.0018) are reported in blue bold.

|  |  | **CTRLs** | | | **CHD** | | |
| --- | --- | --- | --- | --- | --- | --- | --- |
|  |  | BMI  kg/m^2^ | Weight  (Kg) | Waist (cm) | BMI  kg/m^2^ | Weight  (Kg) | Waist (cm) |
| **L-Gal 9 SAT** | r  p | 0.444  0.050 | 0.303  0.194 | 0.338  0.171 | 0.298  0.042 | 0.147  0.323 | 0.165  0.291 |
| **L-Gal 9 PAT** | r  p | 0.182  0.430 | 0.209  0.364 | 0.355  0.148 | 0.092  0.522 | 0.02  0.91 | 0.086  0.572 |
| **L-Gal 9 EAT** | r  p | 0.503  0.020 | 0.557  0.009 | 0.643  0.003 | -0.122  0.390 | 0.097  0.585 | 0.011  0.941 |
| **CD206 SAT** | r  p | 0.490  0.021 | 0.369  0.091 | 0.321  0.18 | **0.520**  **<0.001** | **0.484**  **<0.001** | 0.43  0.003 |
| **CD206 PAT** | r  p | 0.438  0.047 | 0.377  0.092 | 0.403  0.097 | 0.318  0.022 | 0.218  0.12 | 0.28  0.056 |
| **CD206 EAT** | r  p | 0.265  0.234 | 0.247  0.269 | 0.363  0.126 | -0.098  0.489 | -0.055  0.698 | 0.133  0.374 |
| **NOS2**  **SAT** | r  p | -0.309  0.355 | -0.068  0.841 | 0.238  0.57 | -0.004  0.986 | -0.006  0.975 | -0.074  0.725 |
| **NOS2**  **PAT** | r  p | -0.566  0.044 | -0.367  0.218 | -0.36  0.277 | 0.020  0.916 | -0.004  0.981 | 0.192  0.319 |
| **NOS2**  **EAT** | r  p | 0.012  0.965 | 0.315  0.235 | 0.388  0.171 | -0.290  0.097 | -0.242  0.168 | -0.149  0.422 |

**Supplementary Table 3.** *Correlations between L-Gal 9, CD206 and NOS2 circulating levels and serum lipids and CRP in CHD patients.* Significant correlations after Bonferroni`s correction (6 comparisons; p < 0.008) are reported in blue bold.

| **CHD** | | | | | | | |
| --- | --- | --- | --- | --- | --- | --- | --- |
|  |  | BMI  kg/m^2^ | TC  (mmol/l) | HDL-C (mmol/l) | LDL-C (mmol/l) | TG (mmol/l) | hsCRP (mmol/l) |
| **L-Gal 9**  (ng/ml) | r  p | 0.089  0.531 | 0.218  0.129 | **-0.458**  **<0.001** | 0.316  0.025 | **0.525**  **<0.001** | **0.369**  **0.007** |
| **CD206**  (ng/ml) | r  p | -0.219  0.153 | -0.17  0.915 | -0.091  0.565 | -0.14  0.928 | -0.132  0.403 | -0.105  0.496 |
| **NOS2**  (pg/ml) | r  p | -0.166  0.271 | 0.106  0.494 | 0.033  0.833 | 0.051  0.744 | 0.094  0.542 | 0.023  0.878 |

# Supplementary Figures Caption

**Supplementary Figure 1.** *Significant correlations between L-Gal9, CD206 and NOS2 expression in the AT compartments of CHD patients*

**Supplementary Figure 2.** *Significant correlations between L-Gal 9 and CD206 expression within SAT in CTRLs (****A****) and within EAT in CHD patients (****B****).*
